# Supplementary material for: Tead1a Initiates Transcriptional Priming Through the TEAD1a/YAP‐Notch1‐Spi1/Cebpα Axis to Promote Neutrophil Fate
Source: Adv Sci (Weinh). 2025 Aug 28;12(41):e05441. doi: 10.1002/advs.202505441 (PMC12591127; doi:10.1002/advs.202505441)
Supplement: Supplementary file 4 — Supplementary Dataset [file ADVS-12-e05441-s004.zip › 3-3.zip!/3-3/02-3.cluster/cluster8/Enrich/ego_MF.pdf]

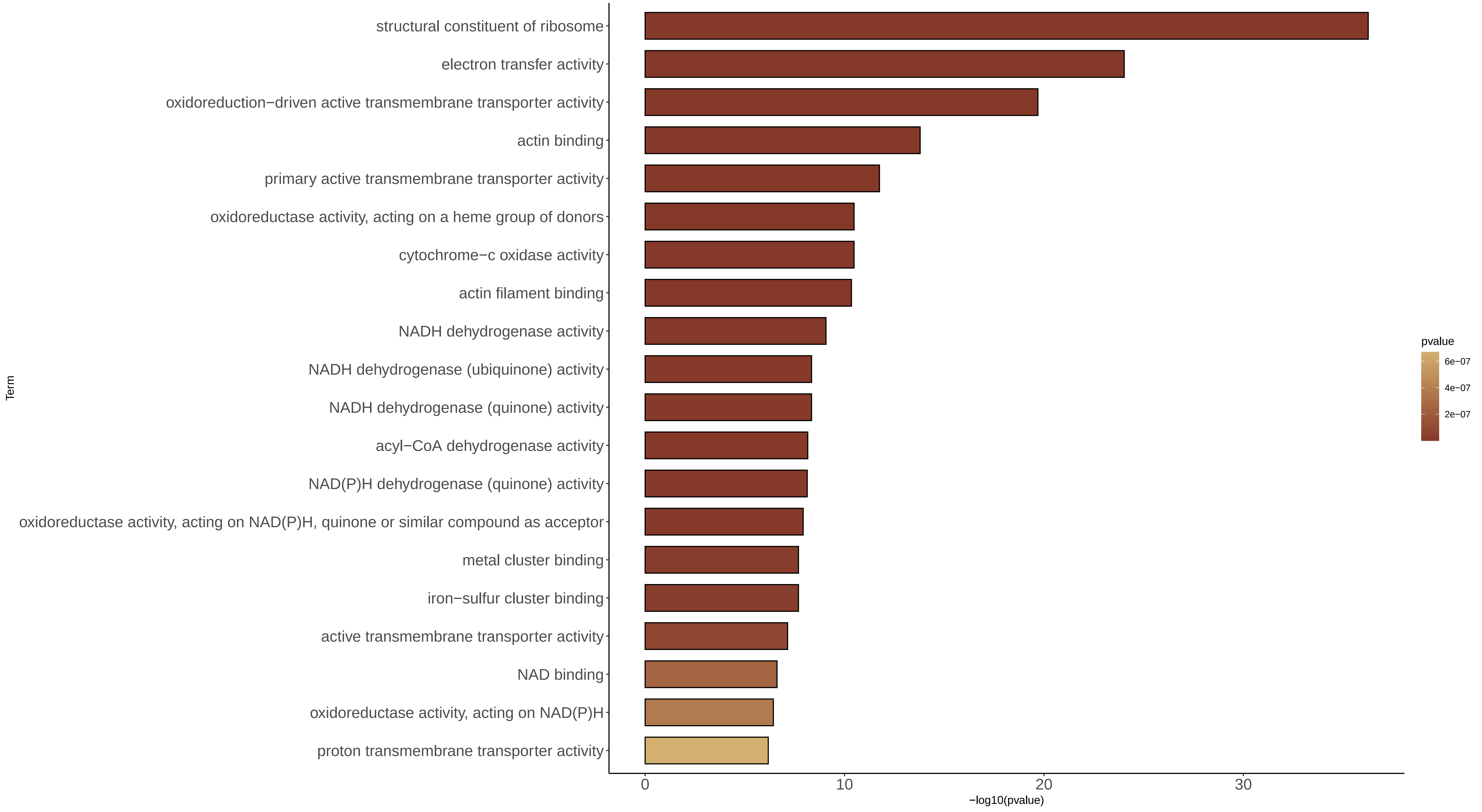
structural constituent of ribosome
electron transfer activity
oxidoreduction−driven active transmembrane transporter activity
actin binding
primary active transmembrane transporter activity
oxidoreductase activity, acting on a heme group of donors
cytochrome−c oxidase activity
actin filament binding
NADH dehydrogenase activity
NADH dehydrogenase (ubiquinone) activity
NADH dehydrogenase (quinone) activity
acyl−CoA dehydrogenase activity
NAD(P)H dehydrogenase (quinone) activity
oxidoreductase activity, acting on NAD(P)H, quinone or similar compound as acceptor
metal cluster binding
iron−sulfur cluster binding
active transmembrane transporter activity
NAD binding
oxidoreductase activity, acting on NAD(P)H
proton transmembrane transporter activity
Term
0
10
20
30
−log10(pvalue)
pvalue
6e−07
4e−07
2e−07
